# Theta Band Zero-Lag Long-Range Cortical Synchronization via Hippocampal Dynamical Relaying

**DOI:** 10.1371/journal.pone.0017756

**Published:** 2011-03-08

**Authors:** Leonardo L. Gollo, Claudio R. Mirasso, Mercedes Atienza, Maite Crespo-Garcia, Jose L. Cantero

**Affiliations:** 1 IFISC, Instituto de Física Interdisciplinar y Sistemas Complejos (CSIC-UIB), Campus Universitat de les Illes Balears, Palma de Mallorca, Spain; 2 Laboratory of Functional Neuroscience, Spanish Network of Excellence for Research on Neurodegenerative Diseases (CIBERNED), University Pablo de Olavide, Sevilla, Spain; University of Maribor, Slovenia

## Abstract

Growing evidence suggests that synchronization among distributed neuronal networks underlie functional integration in the brain. Neural synchronization is typically revealed by a consistent phase delay between neural responses generated in two separated sources. But the influence of a third neuronal assembly in that synchrony pattern remains largely unexplored. We investigate here the potential role of the hippocampus in determining cortico-cortical theta synchronization in different behavioral states during motor quiescent and while animals actively explore the environment. To achieve this goal, the two states were modeled with a recurrent network involving the hippocampus, as a relay element, and two distant neocortical sites. We found that cortico-cortical neural coupling accompanied higher hippocampal theta oscillations in both behavioral states, although the highest level of synchronization between cortical regions emerged during motor exploration. Local field potentials recorded from the same brain regions qualitatively confirm these findings in the two behavioral states. These results suggest that zero-lag long-range cortico-cortical synchronization is likely mediated by hippocampal theta oscillations in lower mammals as a function of cognitive demands and motor acts.

## Introduction

Synchronization is an astonishing omnipresent collective phenomenon occurring at any scale, ranging from subatomic to astronomical scales. Synchronization requires the coordination of systems to operate at unison. Synchronized activity has been observed, e.g., in the brain between neurons, in the heart, between laser systems, fireflies and many other natural and manmade systems [1], [2].

The presence of delays has been shown to play a critical role in dynamical systems [3]–[10]. Particularly for neuronal systems, non-negligible delays have been argued to shape spatiotemporal dynamics [11] and to facilitate synchronization [12]–[15]. After extensive theoretical and experimental works the function of synchronization is not yet fully understood but is becoming gradually improved.

Synchronization by neural oscillations contributes to the formation of functional circuits at different spatial scales through a broad range of frequencies [16]–[21]. Specific patterns of neural synchronization have been largely associated with perceptual, motor skills, and higher cognitive functions, providing insights into how large-scale integration can be assisted by oscillatory codes in the mammalian brain [22]–[27]. The phase relationship of synchronized elements has been further suggested as a critical mechanism for the efficiency of such information exchange between neurons located in distant brain regions [28], [29].

*In vivo* and *in vitro* experiments suggest that zero-lag neuronal synchrony occurs in the brain even in the presence of large axonal conduction delays [30]–[32]. From a theoretical viewpoint, modeling zero-lag synchronization in long delayed systems has typically been a challenging task, and different mechanisms have been proposed to account for this phenomenon [33]–[35]. More recently, Fischer et al. [36] introduced a novel and robust concept of synchronization via dynamical relaying. This concept suggests that two distant neuronal populations are able to synchronize at zero or near zero time lag if a third element acts as a relay between them. This relay symmetrically redistributes its incoming signals between the two other regions. Interestingly, this mechanism has proven to be remarkably robust for a broad range of conduction delays and cell types [37]. A requirement for achieving synchrony without time lag is that the involved brain generators oscillate endogenously or by coupling with other areas. In this context, the thalamus has been recently proposed as a pivotal region generating isochronal gamma range synchronization between distant cortical areas by means of the dynamical relaying mechanism [38].

Although the main generators of theta oscillations are located in the hippocampus, this oscillatory activity has been observed in many cortical and subcortical regions [39]–[41]. However, none of them are capable of generating theta activity on their own [19] despite some models of recurrent excitation predicted the generation of coherent theta oscillations in neocortical networks [42]. Functional coupling between hippocampal and neocortical theta waves have recently been observed in rodents, likely revealing binding of cortico-hippocampal systems modulated by cognitive and behavioral demands [43], [44]. Long-range cortico-cortical synchrony without time lags has been previously reported between areas subserving related functions [30], [45], but the impact of the hippocampus on cortico-cortical theta oscillatory dynamics has been unexplored to date. We hypothesize that if the hippocampus acts as a dynamical relaying center connected to distant regions of the neocortical mantle, then the hippocampus might induce zero-lag synchronization between long-distance cortical regions where theta oscillations do not appear prominently.

The present study tests this hypothesis by modeling local field potentials (LFP) arising from the combined dendritic activity of a large number of neurons in the hippocampus and two distant cortical areas in mice either during spontaneous motor exploratory behavior (active) or motor quiescence (passive). We found that zero-lag synchronization between both cortical regions was mediated by prominent theta oscillations in the hippocampal formation in the two behavioral states, although it was enhanced during motor exploratory state, where the hippocampus has been suggested to play a critical role in sensorimotor integration [46].

## Results

Zero-lag long-range synchronization emerged between the anterior (frontal) and posterior (occipital) cortical regions when the amplitude of theta oscillations was prominent in the hippocampus. The cortico-cortical zero-lag correlation was approximately 45% higher in the active (when exploring) than in the passive state (when quiet), as revealed by our experimental and modeling results. The theta oscillations recorded in the hippocampus (relay element) were delayed by ∼30 ms which is a strong signature of the dynamical relaying phenomenon [36]–[38].

In the following, we show results obtained from numerical simulations and LFP recorded data. We start with analyzing the neuronal population dynamics and show how theta frequency emerges in the system. Next, we simulate synchronization patterns within the neocortical-hippocampal circuit in passive and active states. Finally, we compare the simulations with the experimental data.

### Modeling theta oscillations generated in the hippocampus

We modeled the hippocampus and the frontal and occipital cortices. Each area contains 500 sparse and randomly connected neurons described by the Izhikevich model. This model uses two variables: the membrane potential *v* and a recovery variable *u*, associated with slow ion channels. We assume that, within each area, 80% of the neurons project excitatory synapses (AMPA) and 20% inhibitory synapses (GABA). Synapses are mathematically described in equation (4) (see Materials and Methods section). Each neuron in the hippocampus (cortical areas) is assumed to receive 35 (50) synaptic inputs from randomly chosen neurons of the same area with negligible conduction delay. The connectivity between areas is considered even sparser. Neurons of a given area are innervated by three excitatory synapses with long conduction delays (8–20 ms) from each of the other areas. The ultimate goal of the model is to compare the neuronal activity of the three areas during the active moving and passive quiescent motor behavioral states. The active state is modeled by assuming a ∼6% larger external driving over the hippocampus with respect to the cortical areas. This is obtained by increasing the Poisson rate of the external driving.

**Figure 1:**
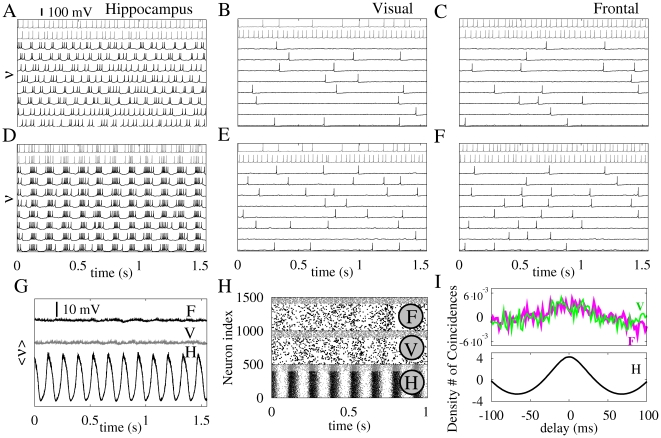
Dynamical characterization of the hippocampus and cortical regions during the generation of theta oscillations. Panels A, B and C show the voltage v time traces of 10 randomly chosen neurons (8 excitatory in black and 2 inhibitory in grey) of each population in the absence of local and long-range connections. Panels D, E and F show the same time traces of neurons locally connected within each population. Panel G shows the ensemble average voltage v of each area: Frontal cortex (F), Visual cortex (V) and the Hippocampus (H). Panel H shows raster plots. Panel I shows an average number of coincident spikes of neuron pairs of the same population, obtained from the auto-correlation function and subtracted from the mean number of coincidences over the delay window. The upper figure in panel I displays cortical groups while the bottom figure stands for the hippocampus. External driving to each neuron is given by 100 independent excitatory neurons spiking according to a Poisson distribution with average rate r = 16.3 Hz.

The capacity of the rodent hippocampus to generate theta oscillatory activity is well documented [19], [47]–[49]. Our model assumes that the hippocampus is mainly composed of neurons operating in a burst regime whose activity is modulated by slow theta oscillations (frequency range from 6.5–7.5 Hz) and an interspike frequency of 35–45 Hz. We consider that most neurons in the cortical areas fire in the regular spiking regime. Diversity within each population is added to the internal neuronal parameters of the model (see Materials and Methods section). The spiking activities of the different regions are illustrated in Fig. 1. Time traces of ten randomly chosen neurons, eight excitatory (black) and two inhibitory (gray), are plotted in Figs. 1 A–F, corresponding to the hippocampus (A & D) and the visual (B & E) and frontal (C & F) cortical regions, respectively. In panels A–C, neurons are completely disconnected from each other, at both global and local levels. The lack of correlation between neuronal activities was due to the assumed random initial conditions. When neurons are coupled within each population, keeping the inter-population coupling strength equals to zero, hippocampal neurons start to synchronize, as displayed in panel G. This synchronization pattern gives rise to a theta oscillation reflected in the time evolution of the average membrane potential shown in panel G. On the contrary, cortical neurons do not fire synchronously, as illustrated in panels E and F, resulting in an almost flat time trace of the average membrane potential (panel G). This behavior is also evident in the raster plots shown in panel H. To determine the level of synchronization, we computed the auto-correlation function as the number of spike coincidences of neurons belonging to the same population (bins of 2 ms), subtracted from the number of coincidences expected by chance, as shown in Fig. 1 I. A coherent behavior was observed in the hippocampus, but not in the cortical areas.

### Dynamical relaying in the theta range

**Figure 2:**
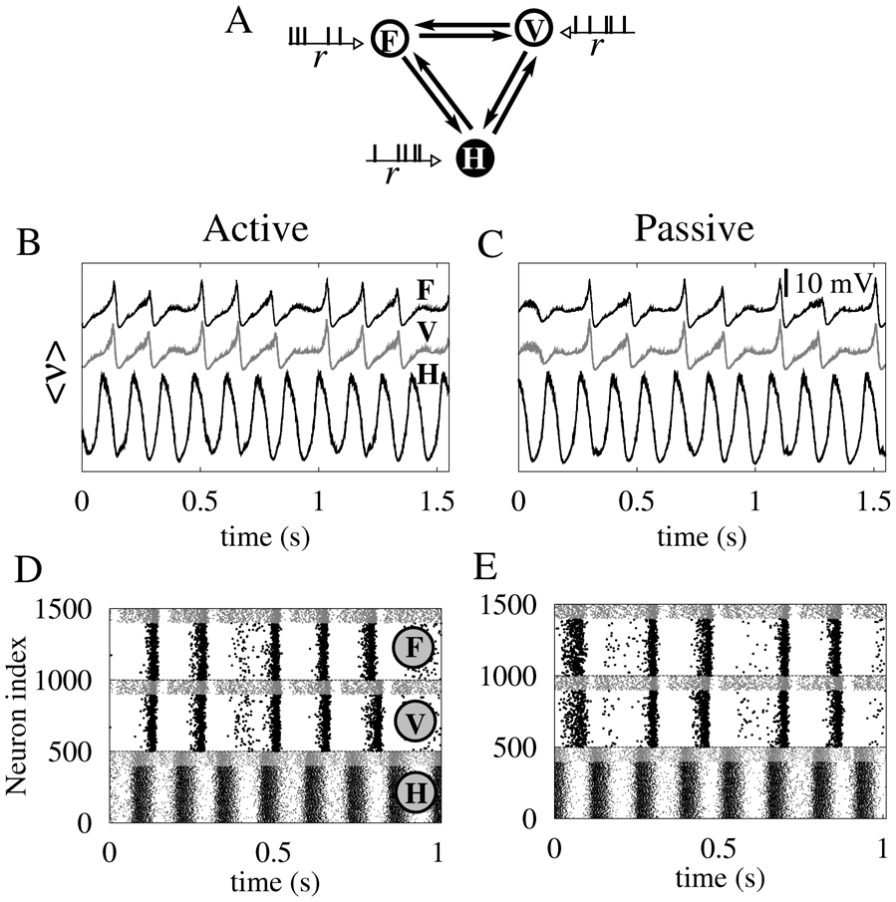
Modeling neuronal dynamics underlying passive and active behavioral states. Panel A represents the simple motif connecting the brain regions F, V and H. Each neuron is driven by an independent Poisson process of rate r = 16.3 Hz (r = 15.4 Hz) for the active (passive) state. In panels B and C, the ensemble average voltage for the passive and active states are plot respectively. Panels D and E include the corresponding raster plots.

Results from our model predict the emergence of zero-lag synchronization between frontal and occipital cortices, but not between the cortical regions and the hippocampus when the long-range connection is switched on (this will be discussed later). The proposed reduced model, as will be shown below, captures the main features observed experimentally. A large-scale integration is maintained by interconnecting the cortical populations and the hippocampus via long-range fibers, with large conducting delays. Our simple motif depicted in Fig. 2 A, is sufficient to reproduce the two behavioral states. In the model, the difference between the two states is on the Poisson rate of the external driving. Both states present zero-lag synchronization between cortical areas as revealed by the mean-voltage time traces represented in Figs. 2B and C, as well as in the raster plots (Figs. 2D and E). In the network, cortical activity becomes locally synchronized due to theta oscillations generated in the hippocampus, when both the internal and long-range connections between the different areas are active. Raster plots also reveal the presence of two different groups of neuronal activity in each area: one of excitatory neurons (black) and the other of inhibitory ones (gray). Unlike neural assemblies in the two cortical areas that synchronize at zero-lag, neural activity in the hippocampus was phase locked, but shifted with the activity in cortical neurons.

### Large-scale motifs

**Figure 3:**
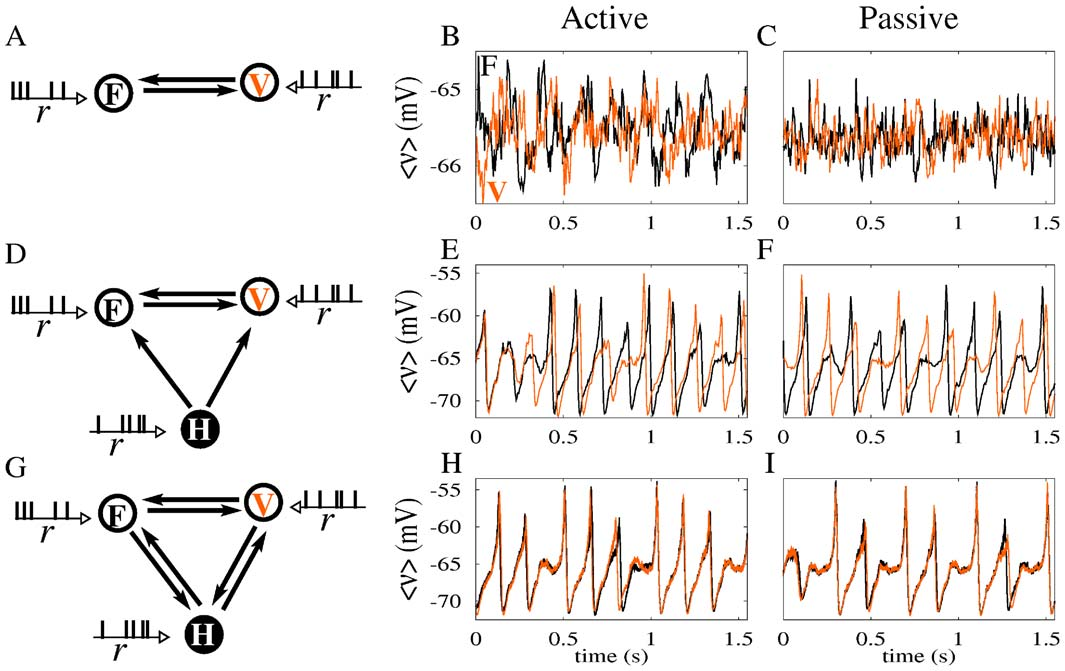
Zero-lag cortico-cortical synchronization for different motifs. Simulation results for the ensemble average voltage of the cortical regions are shown for two external drives corresponding to the active (r = 16.3 Hz) and passive (r = 15.4 Hz) states. Regardless of the behavioral state, we found that the two cortical areas (frontal and visual) do not synchronize at zero-lag when mutually connected without the hippocampal relay (panels A–C). Neither we observed zero-lag synchronization when only the hippocampus drives them (panels D–F). The cortical feedback to the hippocampus (as depicted in panel G) is critical to promote zero-lag cortico-cortical synchronization, as depicted in panels H and I.

Our choice of motif is not arbitrary. From a physiological point of view, recurrent connections among the three involved areas are expected. From the modeling point of view other options could be considered. One possibility is to couple bidirectionally only the two cortical areas, as suggested in ref. [33]. However, in this scenario the out-of-phase solution is the one that appears more often [37]. Moreover, for our parameter values, the two areas do not synchronize (Figure 3 A–C). It is worth stressing that theta oscillations are not observed either in these cortical areas. We have also tested a motif with unidirectional coupling between the hippocampus and the cortices, keeping the two cortical regions bidirectionally connected. As shown in Figure 3 D–F, this motif does not yield zero-lag synchronization among the cortical areas when using the same parameter values. The motif that yields the most robust results is the one chosen in the present study, as depicted in figure 3 G–I.

### Zero-lag synchrony is enhanced during motor exploratory behavior

The reduced model proposed here is justified due to the remarkable equivalence with the experimental data in neocortical-hippocampal neuronal systems during both behavioral states. Although our simulations might only reveal a keen difference for the two states, we demonstrate that noticeable differences are present. With both simulated and experimental data, we proceeded as follows. First, the LFP time traces (for the experimental data) and the ensemble average membrane potential (in the simulations) were filtered around the dominant frequency of theta oscillations recorded in the hippocampus (6.5–7.5 Hz). Next the cross-correlogram of the resulting signals of two different areas was performed within a 300-ms window with delays varying from −300 to 300 ms. The time series were shifted by 50 ms to account for the experimental data variability; the procedure was repeated to cover the 60 s time series. The delay corresponding to the maximum of each cross-correlogram window reflects the best suitable coupling delay between the two areas. This delay was used to compute a normalized peak density of the sliding window cross-correlogram. The result represents the probability of finding the best coupling between different areas occurring at a given time delay.

**Figure 4:**
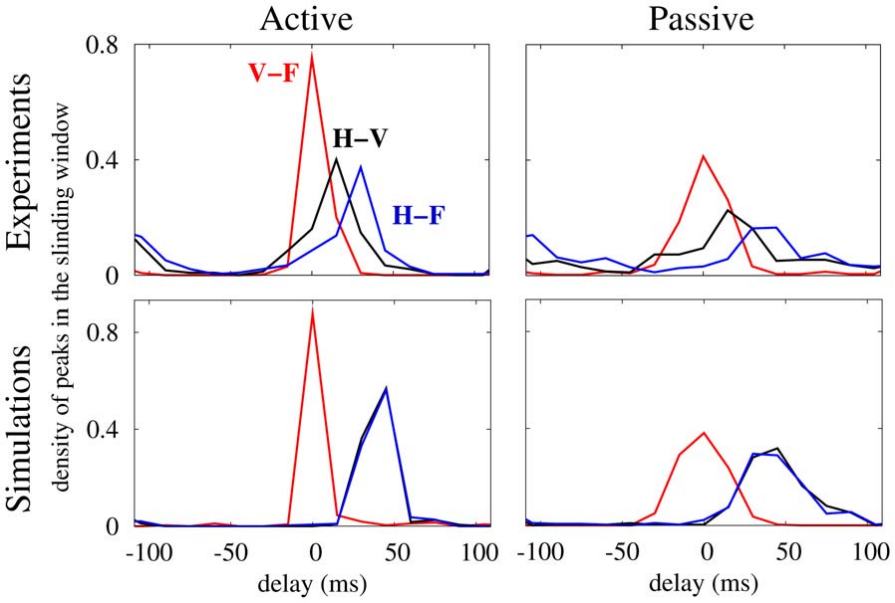
Spatio-temporal synchronization obtained from the experimental and numerical data. We plot here the density of spikes in the sliding window of filtered time series cross-correlation (see Materials and Methods section). The window has 300 ms length and is shifted by 50 ms steps and analyzed over the 60-s of continuous artifact-free LFP recordings for each behavioral state and animal (n = 4), separately. Results are normalized in a frame of −110 to 110 ms. Experimental data correspond, in this example, to an individual mouse, although other mice presented qualitatively similar results. Simulations show high agreement with experimental results for both active and passive behavioral states.

**Figure 5:**
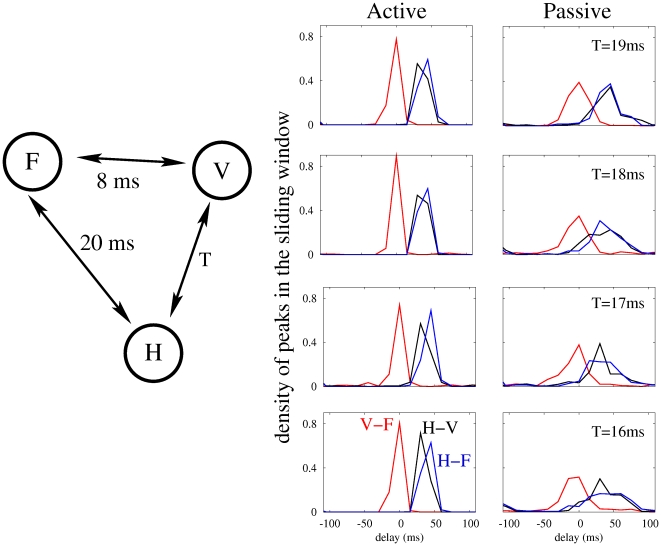
Effects of an asymmetric delay time in the inter-population couplings. If the delay time between the hippocampus and the visual area (T in the figure) is slightly different from that between the hippocampus and the frontal area (20 ms), the maxima of the cross-correlations between the hippocampus and the cortical areas become different, as shown in the experiments (Fig. 4, upper panels).

Following this procedure, we compared the simulated and experimental data for the two behavioral states. A wider and less precise phase locking in the passive condition was observed in both cases. Results in the active state appeared to be more coherent, with higher values of cross-correlograms (Fig. 4). In particular, the two cortical areas were mostly synchronized at zero-lag whereas the hippocampus was typically delayed by 15–30 ms in the active state, and by 15–45 ms in the passive state. The maximum correlation with zero-lag occurred with a ∼45% larger probability in the active state than in the passive state, the latter showing a larger variability in its activity pattern [50]. Synchronization levels between the hippocampus and the cortical areas were also more consistent during active exploration when compared to motor quiescence. Simulations were in remarkably good agreement with our experimental LFP recordings. However, cross-correlograms between the hippocampus and the cortical areas peaked at the same time delay value in the model due to the symmetry assumed in the conduction delays between these areas. We obtained even closer results to the experimental ones in the simulations when considering asymmetric conduction time delays (of the order of few ms) between the hippocampus and the cortical areas (Figure 5).

## Discussion

Although a large body of studies has evaluated the hippocampal-neocortical circuitry underlying theta oscillations [44], [51]–[65], the mechanisms responsible for inducing coherent activity in these regions remain elusive to date [65]. The present study gives a step further by suggesting that these interactions may facilitate communication between distant cortical regions. By borrowing concepts from the dynamical relaying framework, we have studied the impact of hippocampal theta oscillations on cortico-cortical functional coupling in mice during motor quiescent, and while actively exploring the environment. Modeling results showed that zero-lag synchronization between distant cortical regions increased simultaneously with hippocampal theta oscillations in both behavioral states, although cortico-cortical coherence was mainly enhanced during motor exploratory behavior. LFPs recorded from the same brain regions and during the same behavioral states qualitatively confirmed these results. Overall, these findings suggest that the observed zero-lag cortico-cortical synchronization is likely modulated by the hippocampus in lower mammals as a function of cognitive demands and motor acts.

### The role of hippocampal theta oscillations in long-range synchronization

The numerical results obtained from the simple model suggest that theta oscillations are critical for a long-range integration between the hippocampus and the cortical areas, especially when the animal is exploring the environment. We speculate based on the dynamical relaying mechanism that theta oscillations should participate if the hippocampus acts as the relay station that putatively facilitates zero-lag synchrony between distant cortical areas. Interestingly, our results suggest the possible coexistence of dynamical relaying in different frequency bands, for example in a gamma range [37], which could be mediated by the thalamus [38] or other cortical areas [66]. A better understanding of the synchronization in distinct frequency bands is however necessary.

### Dynamical relaying and phase relation

A typical fingerprint of the dynamical relaying mechanism in neuronal systems connected via significant delays is the zero-lag synchrony coexisting with the out of phase synchrony between the relay element and the other two areas [36]–[38]. The novelty of our study with respect to others lies in the inclusion of the occipital cortex in addition to the frontal cortex and the hippocampus. The occipital cortex represents the major source of visual inputs to the hippocampus, and is a key cerebral structure for the formation of spatial memories. Evidence shows that theta-burst stimulation of the thalamocortical pathways leads to a long-term enhancement of granule cell excitability in the hippocampus, preceded by a concurrent potentiation of the visual cortex response. The theta power in the dentate gyrus increases after tetanization-driven high-frequency rhythms in V1. This sequence of events has been suggested to facilitate the induction of synaptic plasticity in the hippocampus of the freely behaving rat [67]. Theta oscillations recorded over posterior neocortical regions during wakefulness have been further postulated as reliable markers of the homeostatic process of sleep regulation in the rat, suggesting that theta waves might have independent cortical generators over the parieto-occipital regions [68].

It is broadly accepted that hippocampal theta oscillations play a crucial role in sensorimotor integration [46] and memory formation [26], [69]. For this endeavor, a precise spiking time is needed. In the context of theta rhythms, the oscillatory phase coupling has recently been proposed to enhance the efficiency of spike-time dependent plasticity [70]. The coordination of neuronal assemblies over distant regions could be critically dependent on the increased oscillatory phase coupling [71], playing a role in the cortico-hippocampal circuit for memory formation. For both sensorimotor integration and memory formation, the hippocampus requires inputs from other regions typically involved in the automatic and voluntary control of attention. Accordingly, memory recollection has been supported by a distributed synchronous theta network including the prefrontal, mediotemporal and visual areas [72]. Based on our findings, we speculate that an enhancement of long-range cortico-cortical synchronization patterns mediated by the hippocampus might facilitate the integration of these top-down and bottom-up control mechanisms of attention.

### Local field potentials recorded from hippocampus and neocortex: the role of volume conduction

Zero-lag synchronization between cortical regions simultaneously to hippocampal theta oscillations could be due to hippocampal-volume conducted theta. Although concerns about volume conduction are significant in the present study, converging evidence also points against this possibility. For instance, Katzner and colleagues [73] found that the major part of the LFP recorded signal (>95%) spreads within 250 µm from the recording electrode, suggesting that the origin of LFPs is more local than often recognized. Moreover, as recently reviewed by Pesaran [74], simultaneous LFP recordings have been extensively used to evaluate the relationship between distant areas including, for instance, the prefrontal and visual cortices [75], the prefrontal and parietal cortices [76] or the hippocampus and the prefrontal cortex [51].

Theta waves recorded in the frontal cortex could be volume-conducted from the olfactory bulb rather than intrinsically generated in the frontal region. Although this hypothesis is conceivable, previous studies have provided strong evidence of theta synchronization patterns between the frontal cortex and the hippocampus [51]. Due to the course-grained nature of our experimental data, we do not have access to the individual neuronal spike time. However, after a filtering procedure it becomes clear that the hippocampus is delayed with respect to the cortical areas.

### Final remarks

We have studied the occurrence of zero-lag synchronization between distant cortical regions. Using a simple model where two cortical areas are both directly connected through the hippocampus we find that the activities in these regions become synchronized in the theta range in freely behaving mice. Our results suggest that the hippocampus might act as a relay element that mediates zero-lag synchronization between the cortico-cortical regions, during active and passive behavioral states. Simulated and experimental data showed that this zero-lag synchronization between two distant remote cortical regions occurs simultaneously with prominent theta oscillations in the hippocampus in both behavioral states, but it is significantly enhanced during exploratory motor behavior. These findings could provide an alternative explanation to the observed zero-lag relationship between distant cortical regions by hippocampal theta.

## Materials and Methods

### Modeling theta synchronization in large-scale systems

We aimed at modeling theta synchronization patterns of the hippocampus, and the frontal and visual cortices supporting the emergent coherent behavior associated to spontaneous exploratory motor behavior and motor quiescence, separately. To this end, we considered three neuronal populations composed of 500 randomly connected neurons, 80% excitatory and 20% inhibitory, with excitatory innervating monosynaptic pathways linking any two of the three regions. We modeled excitatory and inhibitory neurons of the two cortical areas with the following set of equations [77], [78]:
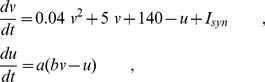
(1)where *v* is the neuron's membrane potential, *u* is the recovery variable that accounts for the K^+^ and Na^+^ ionic currents and *I_syn_* the total synaptic current. When the membrane potential reached the 30 mV value, *v* is reset to *c* and *u* to *u+d*. For excitatory neurons, we take the parameters (*a,b*) = (0.02, 0.2) and (*c,d*) = (−65,8)+(12,−6) σ^2^, where σ is a uniformly distributed random variable within the interval (0,1). According to this distribution, cortical excitatory neurons operate in the regular spiking, in intrinsically bursting or chattering modes [79]. For inhibitory neurons, we assume the parameters (*a,b*) = (0.02, 0.25)+(0.08,−0.05) σ and (c,d) = (−65,8). These parameter values correspond to fast spiking and low-threshold spiking firing modes. With similar computational costs, excitatory neurons of the hippocampus are described with a slightly modified set of equations, specifically calibrated to reproduce the hippocampal CA1 pyramidal neurons dynamics [78]:
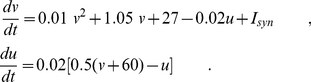
(2)


In this case, when *v* reached the value 40 mV, *v* and *u* are reset as described previously, the parameters are (*c,d*) = (−65,50)+(15,10)σ. This choice favors the bursting mode rather than the regular spiking regime [78], [80]. Inhibitory neurons of the hippocampus are also modeled with the set of equations (1), using identical parameters as for inhibitory neurons of the cortical regions. Anyway, we have checked that different distributions of parameters yielded similar results. Each neuron receives the same number of synapses from randomly selected neighbors of the same population (50 for the cortical populations which means a 10% connectivity, and 35 for the hippocampus, i.e., with a 7% of connectivity), and three long-range excitatory synapses from excitatory neurons randomly selected from the other populations. The local connectivity is composed of both excitatory and inhibitory synapses depending on the neuron type. Excitatory neurons project excitatory synapses and inhibitory neurons project inhibitory synapses. Each region corresponds to a coursed grained brain region, which is recurrently connected. Such connectivity (depicted in Fig. 2 A) composes a bidirectional triangular motif of the three regions of interest. The simple motif connection is satisfied only on the large scale. At the neuronal level, the connectivity is different.

The synaptic current is given by:
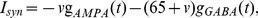
(3)and the synaptic dynamics are described by:
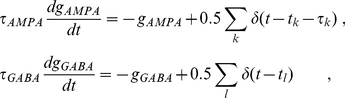
(4)where δ stands for the Dirac delta function. The summation over *k* (*l*) stands for excitatory (inhibitory) neighbor contributions. *t_k_* (*t_l_*) is the time at which excitatory (inhibitory) firings occur in presynaptic neurons. Conduction delays τ_k_, associated to excitatory long-range connections, are assumed to be 8 ms for cortico-cortical connections and 20 ms for the connections between the cortical regions and the hippocampus. Synapses are modeled by exponential decay functions [81] with time constants τ_AMPA_ = 5.26 ms for excitatory and τ_GABA_ = 5.6 ms for inhibitory synapses (other decay times produced qualitatively similar results). Each population is subject to an external driving given by independent Poisson spike trains, resulting from 100 excitatory neurons, at rate *r* = 15.4 Hz on each neuron in the passive state, and 16.3 Hz in the active state. The equations were integrated with the Newton method with time steps of 0.05 ms.

When modeling neuronal dynamics is always desirable to use simple, but biologically realistic, models. The non-linear equations used in this study are rather simple but allow at the same time for some flexibility. They were derived and adjusted to fit certain behaviors: regular spiking, intrinsically bursting, chattering modes, fast spiking or low-threshold spiking. The population of spiking neurons approach gives rise to a robust dynamic and the possibility to compare with experiments at different spatial scales. It has been shown suitable for studying general dynamical patterns [82], [83] and zero-lag synchronization [37], [38]. Utilizing the same neuronal model with a different set of parameters, arbitrary but specifically calibrated to reproduce the diverse dynamics of existing neurons, the isolated hippocampus generates theta rhythms as experimentally demonstrated [49]. In contrast, isolated cortical areas do not have prominent theta, however, the emergence of these oscillations witnessed by the presence of the hippocampal relay. Parameters responsible for population and inter-population couplings were chosen to reproduce the dynamical regimes observed in the experiments. This set of parameters is not considered unique. Canonical models are also expected to be useful to study the dynamical relaying mechanisms with the advantage of being more comprehensible although less biologically plausible.

### Modeling theta synchronization in different behavioral states

In our model, differences between active and passive states are attributed to the rate of the uncorrelated external drives. We assume that when the animal is performing the exploratory task, not only the regions of interest are active but also many other regions contribute. On the contrary, during motor quiescence, we assume that a smaller number of regions are involved, and consequently the total external driving is considerably weaker. The possibility that an increased background activity accounts for a model transition is sustained by the increased scale-free activity found in the cortex during cognition [84], and is also consistent with the proposal that the external driving over the thalamus is a key element to control the engaging and disengaging of a zero-lag cortical synchronization [38]. The dynamical relaying mechanism is remarkably robust to reproduce the observed patterns, although similar results could also be reproduced in other ways. We have checked, for instance, that using a correlated external driving or by changing the coupling strength among neurons (either for intra-population connectivity, for inter-population synapses, or for both of them) yielded qualitatively similar results (data not shown).

### Synchronization measurements from correlation function

Our results described theta synchronization patterns between the cortical areas and the hippocampus during different behavioral states in the alert animal. We used correlation analyses to determine the level of synchrony of the hippocampus-neocortical and cortico-cortical networks, separately. Data were analyzed from the time series using both ensemble average voltage and spike time coincidences. The mean voltage of the time series is filtered in the dominant frequencies of the spectrum corresponding to the theta band (from 6.5 to 7.5 Hz), and the cross-correlation function is computed via a sliding window of 300 ms width, displaced 50 ms from each other over the 60-s of continuous artifact-free LFP recordings for each behavioral state and mouse, separately. The cross-correlation between two areas A1–A2 as a function of the delay *d* is defined as:
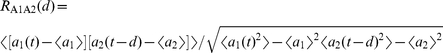
where *a_1_* and *a_2_* correspond to the LFP time series (ensemble average membrane potential over a population) in the experiments (simulations), and the brackets <⋅> stand for the time average computed for each window. The delays corresponding to the maximum peak of the cross-correlation in each window are displayed in a normalized histogram window with times ranging from −110 to 110 ms. Furthermore, in the simulations, the number of spike coincidences is measured from the activity of neurons belonging to the same population (auto-correlation) in 2 s with 50,000 pairs randomly chosen in bins of 2 ms.

### Experimental protocol

All the experiments were carried out according to EU (2003/65/CE) and Spanish (BOE 252/34367-91, 2005) guidelines for the care and use of laboratory animals for chronic experiments. The experimental protocols were previously approved by the Ethics Committee of the University Pablo de Olavide (permit number 07/2). Mice (n = 4, 5 months old) were implanted with electrodes in the CA1 subfield of the hippocampal formation, and in two distant neocortical regions (frontal and occipital cortex) under stereotaxic guidance. The reference electrode was located above the cerebellum (1 mm posterior to lambda on midline). Following experiments, mice were deeply anesthetized with a lethal dose of Nembutal. To verify the electrode placement, sections were mounted on gelatin-coated slides, stained with the Nissl method, dehydrated, and studied with light microscopy.

LFPs were recorded in the animal's home cage with a sampling rate of 200 Hz. 60-s of continuous artifact-free LFP recordings, selected both during exploratory motor behavior (active state) and motor quiescence (passive state) in each animal. The running speed was similar in both groups of mice. The averaged spectral power was estimated by applying the Welch's modified periodogram method (4-s segments, 1 Hz resolution, 50% overlapping, and Hanning windowing) to selected LFP recordings in each LFP derivation. The theta (5–11 Hz) peak frequency was identified as the maximum spectral power value for each cerebral site and animal, separately, by using custom scripts written for Matlab v. 7.4 (The MathWorks Inc., Natick, MA).
